# *In vivo* organ specific drug delivery with implantable peristaltic pumps

**DOI:** 10.1038/srep26251

**Published:** 2016-05-17

**Authors:** Joshua S. Speed, Kelly A. Hyndman

**Affiliations:** 1 Cardio-Renal Physiology and Medicine, Division of Nephrology, Department of Medicine, The University of Alabama at Birmingham, Birmingham, AL 35294, USA.

## Abstract

Classic methods for delivery of agents to specific organs are technically challenging and causes superfluous stress. The current study describes a method using programmable, implantable peristaltic pumps to chronically deliver drugs *in vivo*, while allowing animals to remain undisturbed for accurate physiological measurements. In this study, two protocols were used to demonstrate accurate drug delivery to the renal medulla. First, the vasopressin receptor-2 agonist, dDAVP, was delivered to the renal medulla resulting in a significant increase in water retention, urine osmolality and aquaporin-2 expression and phosphorylation. Second, in a separate group of rats, the histone deacetylase (HDAC) inhibitor, MS275, was delivered to the renal medulla. HDAC inhibition resulted in a significant increase in histone H3-acetylation, the hallmark for histone deacetylase inhibition. However, this was confined to the medulla, as the histone H3-acetylation was similar in the cortex of vehicle and MS275 infused rats, suggesting targeted drug delivery without systemic spillover. Thus, implantable, peristaltic pumps provide a number of benefits compared to externalized chronic catheters and confer specific delivery to target organs.

*In vivo* delivery of drugs or other substances such as siRNA to specific organs can be technically challenging. Chronic catheters have been implanted into a number of vessels including the femoral vein^1^, thoracic aorta^2^, portal vein^3^, vena cava^3^, and organs including stomach^4^, brain ventricles^1^, and kidneys^5^,^6^,^7^. Implanting chronic catheters requires a labor-intensive surgery including suturing a metal spring to the back of the animal to protect the catheters used for infusions. Because this requires long catheter lengths and specialized cages to tether the animal, large amounts of drug are needed to adequately prime the tubing, adding excess costs to experiments. Further, a common and severe complication of tethered catheters is biofilm development and infection^8^, which is also experienced by hemodialysis and chemotherapy patients in the clinic^9^. Because of this, chronic catheterization typically requires additional animals to account for technical failures within an experiment. Although these methods have been successful in furthering our understanding of physiology and disease, there is a need to improve on current methods to avoid undue stress and infection that may confound physiological measurements, while maximizing drug delivery to organs of interest.

Subcutaneous osmotic mini-pumps are widely used to deliver agents in rodent models^10^,^11^. However, they are generally, but not limited to, delivery of drugs systemically, are limited to only a single use, and can only deliver extremely small volumes at low infusion rates. The low infusion rates require one to make up drugs and compounds at concentrations that may be outside of the solubility range. In addition, the flow rate of an osmotic mini pump is fixed and once implanted it cannot be stopped and restarted. Therefore, in order to infuse several different solutions over time or generate a dose response, implanting new osmotic mini pumps or using mini pumps with cannulas filled with different solutions separated by bubbles or mineral oil (to prevent mixing)^12^ are required. Recently, a small implantable, programmable, peristaltic pump became commercially available (iPrecio^®^, Tokyo, Japan)^13^ (Fig. 1A). The purpose of this study was to develop an improved method to chronically deliver drugs to specific organs for targeted drug delivery. In these proof of concept experiments, we targeted the medullary region of the kidney because chronic tethered catheter use has been instrumental in our understanding of kidney physiolgy^5^,^6^,^7^.

## Methods

### Animal surgery

All animal use and welfare adhered to the NIH Guide for the Care and Use of Laboratory Animals following a protocol reviewed and approved by the Institutional Laboratory Animal Care and Use Committee of The University of Alabama at Birmingham. Sprague Dawley, 8 week old male rats (225 g) were purchased from Harlan (Indianapolis, IN) and maintained on a 12 h light 12 h dark schedule. Rats were fed a normal salt diet (0.49% NaCl Teklad TD.96208) and allowed water *ad libitum*.

For all animal surgeries, proper aseptic technique was used, and all drapes, supplies, surgery tools and gloves were sterilized. Rats were anesthetized with 2% isoflurane and given an s.c. injection of carprofen (5 mg/kg) and buprenorphine (0.1 mg/kg) to minimize pain post surgery. Just lateral to the left rib cage, hair is shaved, and the skin prepared by three alternative wipes of 10% betadine (Purdue Pharma, Stamford, CT) and 70% ethanol (in water). A small incision is made through the skin and muscle, and the kidney exposed. The adrenal gland of the left kidney was carefully freed from the upper pole of the renal capsule before the renal pedicle is ligated with 5-0 silk suture (Ethicon, Summerville, NJ) and the kidney removed. The muscle was sutured closed with 4-0 prolene suture (Ethicon) and the skin was closed with surgical staples. The area was cleaned with 3% hydrogen peroxide in water, and the incision was given 0.25% Marcaine + 0.5% lidocaine (mixed 50/50). The animal was placed in a clean cage and allowed to recover for 7 days before the pump surgery. Animals were closely monitored after surgery and if they lost more than 20% body weight, appeared lethargic or lost righting ability, they would’ve been euthanized and excluded from the study; however, in this study that was not observed, thus all animals in this study were included.

### Pumps, modification and pump surgery

**Figure 1:**
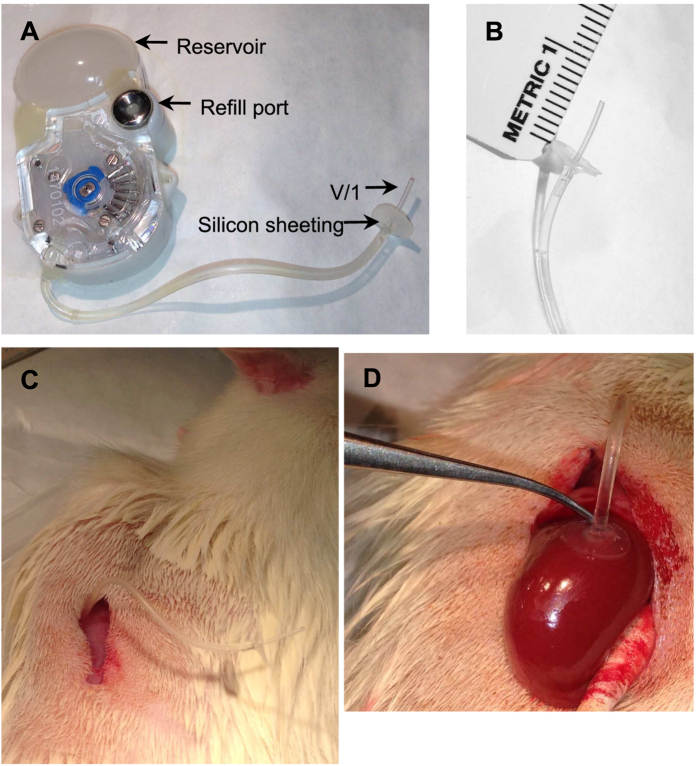
Preparation and insertion of implantable, peristaltic pumps (iPRECIO^®^) into rats for the study. (**A**) The reservoir of the sterile pump is filled through the port, and the catheter is fitted with a silicon sheet and V/1 tubing. (**B**) The V/1 tubing is 8 mm in length. (**C**) The pump fits in a subcutaneous pocket on the dorsal side of the rat. (**D**) the V/1 tubing is inserted into the kidney and adhered.

In this study, implantable peristaltic pumps (iPrecio^®^ model smp-200, Tokyo, Japan) were modified for delivery of drugs to the renal medulla. These pumps are 38.7 × 19.2 × 9.7 mm and emptied weight is 7.9 g. The reservoir holds a maximum volume of 900 μl of reagents. The pumps can be programmed to flow at rates from 1 ± 0.1 μl/h to 30 ± 0.1 μl/h, and before surgery pumps can be programmed to have a constant or variable flow rate depending on the end user’s experimental design. The battery life of the pump depends on the flow rate, but it is predicted to pump for 6 months at 1 μl/h or 1 week at 30 μl/h. In our hands, we have used pumps at 30 μl/h for 30 min followed by 9 μl/h for over 1 month of pumping time. The refill rate will be dependent on the flow rate, so for example at 1 μl/h the pump will need to be refilled in 37 days, while at 9 μl/h it will need to be refilled every 4 days. Currently they cost ~$270/pump. Although they are considered “disposable” we have successfully reused pumps. In the MS275 study (see below) the pumps were new but in the dDAVP study (see below) the pumps were sterilized and reused. The sterilization protocol in listed in Supplementary Note 1. Just prior to surgery, the pumps were programmed to pump at 30 μl/h for 30 min to maintain catheter patency and then 9 μl/h for the remainder of the study. Next the pumps were removed from their packaging, and the catheter cut to 6 cm in length (Fig. 1A). The end of the catheter was modified (for renal medullary specific infusion in this case) by placing a 5 mm circle of silicone sheeting and inserting a small piece of micro medical grade catheter vinyl tubing (V/1) into the catheter. The joint between catheters was sealed and secured by a small drop of superglue (Fig. 1B). The pump was then placed subcutaneously on the back, and sutured into the muscle (Fig. 1C). A small incision was made through the muscles of the abdomen, and the catheter inserted 7–8 mm into the renal capsule and adhered with Vetbond^®^ (Fig. 1D). The muscles were then sutured together and the skin closed with staples. The rats were allowed to recover for 2 days prior to delivery of drugs or saline. Detailed explanation of the surgery is located in Supplementary Note 1 and can be found in the Nature Protocol Exchange.

### Drug delivery

In this study, there were 2 different experimental protocols: 1) Vehicle (saline) compared to the vasopressin receptor-2 agonist, dDAVP (n = 4/ group). 2) Vehicle (30% DMSO in sterile saline) compared to the class 1 histone deacetylase inhibitor, MS275 (n = 5/group). At the start of each protocol, each rat was anesthetized with 2% isoflurane, and the remaining saline in the pump removed from the reservoir by inserting a 27-gauge needle syringe percutaneously and withdrawing. The pump was then filled with either vehicle, 1.1 ng/μl dDAVP, or 1.5 μg/μl MS275 and the rats were placed in a metabolic cage for urine collection. Emptying and refilling of the pumps was all done via a syringe and needle percutaneously without the need to re-open the wound and externalize the pump. The rats were also challenged with high salt diet (4.0% NaCl) during the infusion. dDAVP was delivered at 10 ng/h at a rate of 9 μl/h to the interstitium of the renal medulla for 4 days. This rate of delivery was based upon previous studies using tethered catheters that determined 10 μl/min infusion of 0.9% saline had no significant effect on renal hemodynamics^6^, thus we were well below this delivery rate and don’t anticipate affecting renal hemodynamics. Second, at this rate, the pumps have to be refilled every 96 h, thus limiting the amount of handling incurred by the animals. MS275 was also delivered at 9 μl/h, which resulted in a delivery of 1mg/kg/day. Urine was collected every 24 h for 4 days. Urine samples were spun 1000 g for 10 min, snap frozen and stored at −80 °C until analysis.

### Flow rate Accuracy

Flow rate accuracy was determined *ex vivo* and *in vivo* with new and previous used pumps. For *ex vivo* determination of flow rate accuracy, pumps were programmed (9 μl/h or 30 μl/h), filled with 900 μl of sterile saline, and weighed. The pumps were kept in 200 ml of water in a 400 ml beaker at 37 °C. Over the course of 48 h the pumps were carefully hand dried and weighed 4 different times. For *in vivo* determination, pumps were programmed (listed in Table 1) and implanted as described above. After 12 h or 3 days, the volume remaining in the pump was determined by percutaneously withdrawing the remaining solution. From either the weight (*ex vivo*) or volume (*in vivo*) the volume pumped was plotted against time and linear regression analysis performed. The slopes calculated from these data were compared to the slope of the expected volumes. The % accuracy was calculated as ((computed slope-the expected slope)/expected slope)*100, and compared to the manufacturer’s flow rate accuracy listed in their technical note (http://www.iprecio.com/technology/tabid/147/Default.aspx)

### Western blots and histone extraction

Inner medullae from Protocol 1, were lysed in 10 vols/ wt of lysis buffer + protease inhibitors and phosphatase inhibitor cocktail (Thermo, Waltham, MA) as previously described^14^, and spun at 6,500× g for 10 min to pellet nuclei. The supernatant (herein referred to as lysate) from this spin was used to determine expression of AQP2 and phosphorylated AQP2.

Outer medulla and Cortex from Protocol 2, were lysed with lysis buffer + protease inhibitors, and phosphatase inhibitor cocktail, and centrifuged at 6,500× g for 10 min at 4 °C to pellet nuclei. Histones were then extracted from the nuclear pellet by acid extraction with 5 volumes of 0.2 N hydrochloric acid, overnight at 4 °C. The histone sample was then spun at 6,500 g for 10 min at 4 °C to pellet debris, and the protein concentration of the histone supernatant and original lysate determined by Bradford assay (Quickstart^®^, Biorad, Carlsbad, CA), and samples stored at −20 °C until used in Westerns as previously described^14^,^15^. Antibodies used in the study were Phosphorylated AQP2 S261, S264 or S269 (PhosphoSolutions, Aurora, CO), total AQP2 (SC-9882, Santa Cruz Biotechnology, Dallas, TX), acetylated H3 (#06-599, EMD Millipore, Billerica, MA) and total H3 (06-755, EMD Millipore). Densitometry (arbitrary units) was determined using Image Studio (Li-COR Biotechnology, Lincoln, NE), by an investigator blinded to the treatments.

### Urine and plasma electrolytes

Urine and plasma osmolality were measure by a vapor pressure osmometer (Elitech Group Solutions, Princeton, NJ). Ion selective electrodes were used to determine Na^+^, K^+^ and Cl^−^ concentrations (Easylyte, Medica, Bedford, MA).

### Statistics

All results are reported as mean ± s.e.m. Statistical significance was determined by unpaired, two-tailed Student’s t-test for densitometry of western blot analyses, or repeated measures Two-Factor ANOVA (for drug and time) with Sidak’s multiple comparison (5 comparisons) post hoc for metabolic cage parameters. α = 0.05.

## Results

Flow rate accuracy of the pumps in both an *in vivo* and *ex vivo* setting was determined using a variety of programs in the pumps. As shown in Table 1, our calculated flow rate accuracy for new pumps used *in vivo* programmed to deliver at 9 or 15 μl/h was 1.3% and −3.4% respectively, and was similar to the −2.6% accuracy reported by the manufacturer in an *ex vivo* environment (Table 1). The manufacturer did not provide any accuracy measurements in an *in vivo* setting. Furthermore, the flow rate accuracy was within an overall average ± −2.2% compared to the expected flow rate, suggesting accurate flow rates with new pumps *in vivo*. Reused pumps were also evaluated in *ex vivo* and *in vivo* settings. Again, *ex vivo* and *in vivo* recordings of flow rate accuracy ranges for the various programs were −2.3 to 0.9% of the expected flow rate. Finally, with a subset of the *in vivo* pumps, there were 2 varied flow rate protocols: 1. 30 μl/h for 30 min + 9 ul/h for 3 days, and 2. 15 μl/h for 12 h + 9 ul/h for 12 h. The flow rate accuracy was −5.3 ± 2.5% and −1.3 ± 2.7% respectfully, demonstrating the versatility and accuracy for varied delivery *in vivo* (Table 1). For comparison, the manufacturer of osmotic mini pumps (Alzet, Cupertino, CA) reports that osmotic mini pumps have a variation of infusion rate <10%.

**Figure 2:**
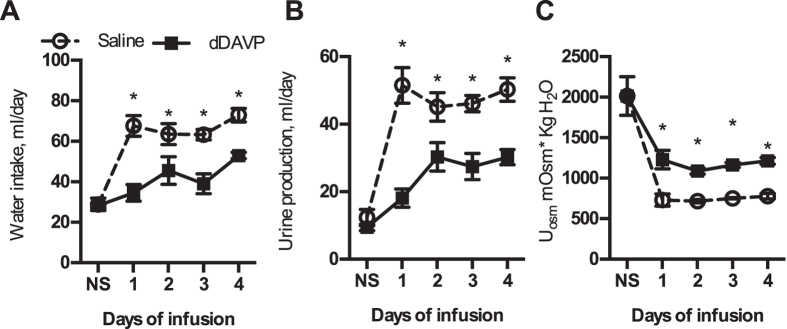
Chronic renal intramedullary interstitial infusion of saline, or dDAVP with implantable, peristaltic pumps. Rats were switched from a normal salt (NS) to high salt diet plus intramedullary infusion. dDAVP intramedullary, interstitial infusion resulted in a significant reduction in (**A**) water intake and (**B**) urine production compared to saline infused control rats (*n* = 4 rats/group, solid line). (**C**) Urine osmolality was significantly increased with dDAVP interstitial infusion, compared to saline infused rats (dotted line). *Represents *P* < 0.05 compared to vehicle infused rats from repeated measures, Two Factor ANOVA analysis.

Compared to vehicle infused rats (n = 4), the rats receiving dDAVP (n = 4) gained significantly more body mass; the change in mass of the saline group was 17.1 ± 1.1 g, while the dDAVP group gained 23.7 ± 1.4 g (*P* = 0.01). In addition, dDAVP treatment caused a significant reduction in water intake (P_group_ < 0.001 P_diet_ < 0.001 P_interaction_ < 0.001 P_subjects matching_ < 0.001) (Fig. 2A) and urine flow (P_group_ < 0.01 P_diet_ < 0.0001 P_interaction_ < 0.0001 P_subjects matching_ < 0.0001) (Fig. 2B) and a significant increase in urine osmolality (P_group_ = 0.01 P_diet_ < 0.001 P_interaction_ = 0.19 P_subjects matching_ = 0.13) (Fig. 2C). Free water clearance for the saline infused group was −1.3 ± 0.06 ml/min and this was significantly greater than the dDAVP infused group −1.6 ± 0.07 ml/min (*P* = 0.02). Food intake, sodium, potassium and chloride excretion and creatinine clearance were similar between the groups (Fig. S1).

**Figure 3:**
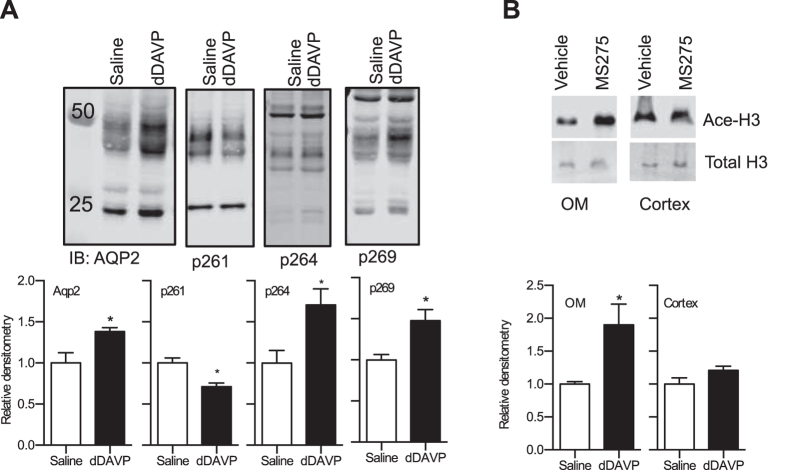
Chronic renal intramedullary interstitial infusion of saline, dDAVP or the histone deacetylase inhibitor, MS275, with implantable, peristaltic pumps. (**A**) Unglycosylated expression (~25KD) and glycosylated AQP2 (gAQP2, >30kD) expression and phosphorylation status at serine 264 and 269 was significantly increased in the inner medulla of rats receiving intramedullary interstitial infusions of dDAVP. AQP2 phosphorylation of serine 261 was significantly reduced in dDAVP treated rats. *represents P < 0.05 compared to saline infused rats (*n* = 4/group) as determined by unpaired, two tailed Student’s *t*-test. (**B**) To demonstrate specificity of the renal intramedullary interstitial infusion, MS275-induced histone 3 acetylation status was determined in outer medullary (OM) and cortical samples (*n* = 5/group). MS275 resulted in a significantly higher H3 acetylation compared to vehicle infused rats in the OM, but there were no significant differences in acetylation status in the cortical samples. *Represents P < 0.05 compared to vehicle infused rats as determined by unpaired, two tailed Student’s *t*-test.

To confirm catheter position, each kidney was dissected at the insertion site (Fig. S2A), and catheter placement was determined by tracing the track left by the catheter (Fig. S2B). Proper placement was seen in all 8 animals, with the catheter track ending at the junction between the outer (OM) and inner medulla (IM) (Fig. S2B). To further confirm vasopressin receptor-2 activation by dDAVP, AQP2 phosphorylation was measured at serine 261, 264 or 269^16^ of the IM (Fig. 3A). AQP2 expression and phosphorylation of serine 264 and 269 were significantly increased and phosphorylation of serine 261 significantly reduced in the dDAVP treated rats, consistent with previous reports^16^ (*P*_*AQP2*_ = 0.03, *P*_*p261*_ = 0.007, *P*_*p264*_ = 0.03, *P*_*p269*_ = 0.02) (Fig. 3A).

Next, to determine if intramedullary interstitial infusion with the pumps leads to systemic or non-specific “spillover”, the histone deacetylase inhibitor, MS275^17^,^18^ was delivered to the renal medulla. The hallmark of HDAC inhibition is hyper-histone H3 acetylation^19^. As shown in Fig. 3B, intramedullary infusion of MS275 resulted in a significant increase in H3 acetylation in the OM of the kidney (*P* = 0.02), but not the cortex (*P* = 0.11).

## Discussion

Chronic catheterization is used in both basic science research with animal models, and in the clinic to deliver drugs and other agents. An unfortunate side effect of chronic catheter use is biofilm accumulation and infection^8^, because they must be externalized to a pump. Thus, there is a need to improve on current methodologies. In the current study, small programmable, implantable peristaltic pumps were used to deliver drugs to the renal medulla. The medulla of the kidney plays a critical role in the regulation of whole body water homeostasis. This is predominantly regulated through the actions of the hormone vasopressin acting on the renal collecting duct to promote water channel (aquaporin-2) expression, phosphorylation and apical surface expression resulting in water retention (anti-diuresis)^16^. In the current study, we present the use of implantable pumps to deliver the vasopressin receptor-2 agonist, dDAVP to the interstitial region of the renal medulla. dDAVP is a well-described agonist used to stimulate aquaporin-2 expression and subsequent water retention in rodents^16^ and to treat diabetes insipidius in humans^20^. As predicted, delivery of dDAVP to the renal medulla led to an increase in all of the hallmark signs of water retention. There were no incidences of infection in either the kidney, or around the pump. This experiment confirms that implantable pumps are ideal for delivery of drugs to specific organs than the classical used of externalized catheters.

Histone deacetylase inhibitors have emerged as novel therapeutic interventions for the treatment of not only cancer, but also cardiovascular and neurological diseases^21^. Specific organ or tumor delivery of HDAC inhibitors may also help prevent undesirable side effects, such as hyponatremia that have been reported with systemic HDAC inhibitor interventions^22^,^23^. In the current study, the Class I HDAC inhibitor, MS275 was used to demonstrate the specificity of delivery that can be achieved with implantable pumps. The gold standard endpoint for confirmation of HDAC inhibition is an increase in histone H3-acetylation. It was confirmed that MS275 infusion of the medulla resulted in an increase in histone H3-acetylation in the outer medulla but not the cortex, indicating the extreme specificity of delivery associated with this method.

In preliminary studies, during optimization of the surgery protocol we found only 3 rats with signs of infection (walling off of the implant with localized infection around pump). After improving sterilization techniques of implants and gas sterilization of tubing and silicone (see Supplementary Note 1), no other signs of infection have been found to date. Although in this study the pumps were only in the animal for 6 days (2 day recovery, 4 day experiment), we have successfully used pumps in animals for 19 days (*personal observation*). Thus, like all survival surgery including chronic catheters, excellent aseptic technique is critical for preventing infection. Furthermore, Tan *et al*.^13^ reported maintaining peristaltic pumps in rats for 30 days, while measuring blood pressure, without any adverse side effects. Tan *et al*.^13^ also demonstrated that when implantable peristaltic pumps are set to the same flow rate as osmotic minipumps to delivery angiotensin II subcutaneously (150 ng/kg/min), implantable peristaltic pumps resulted in a more rapid, and maintained increase in blood pressure compared to osmotic minipumps^13^. Likewise, Kuroki *et al*.^24^ demonstrated very similar results when they compared implantable pumps to osmotic minipumps and externalize catheters over a 2-week infusion protocol (subcutaneous, angiotensin II, 150 ng/kg/min); blood pressure increases were greater with implantable pumps than chronic catheters or osmotic mini pumps.

To conclude, implantable peristaltic pumps provide a number of advantages over current chronic methods. The advantages over osmotic mini pumps include: 1) easy to interchange infusate while pump is implanted, 2) programmable flow rates allowing flexibility of experimental protocol, 3) able to be sterilized and reused in future experiments because of the long battery life (depending on the perfusion rate, batteries can last to pump for 6 months with a 1 μl/h delivery rate), and 4) controlled organ specific delivery, avoiding complications of systemic administration. The advantages over chronic externalized catheters include; 1) no restraining of the animal after surgery, 2) maintenance with standard cages and husbandry with no need for special housing or connection to external pumps, 3) reduced amount of drug needed to fill the pump. These advantages improve the quality of data collected, and because the pump and catheters are implanted subcutaneously there is less risk of infection and loss of catheter patency that is typically observed in models with externalized catheters. However, in experimental designs where small volumes of drug can be delivered at low rates (<10 μl/h) for up to 1 or 2 weeks, osmotic mini pumps are advantageous over implantable pumps. In the present study implantable peristaltic pumps were used to infuse the medullary region of the kidney, but the catheters can easily be modified to infuse blood vessels, other organs or even specific regions of organs such as a particular lobe of the brain, or tumors. This innovative approach has broad applications for studying targeted drug delivery or tissue specific knockdown (e.g. siRNA), and is an extreme improvement over current methods.

## Additional Information

**How to cite this article**: Speed, J. S. and Hyndman, K. A. *In vivo* organ specific drug delivery with implantable peristaltic pumps. *Sci. Rep.* **6**, 26251; doi: 10.1038/srep26251 (2016).

## Supplementary Material

Supplementary Information

## Figures and Tables

**Table 1:** Flow rate accuracy of new and use implantable peristaltic pumps.

	**Flow rate Accuracy**			
	**Manufacturer’s**		**Current Study**	
	***ex vivo***	***ex vivo***	***in vivo***	***in vivo***
Method based on:	mass	mass	volume	volume
pump status	new	used	new	used
Sample size	14–15	6	9	8
1 μl/h	0.19%	n.d,.	n.d.	n.d.
9 μl/h	n.d.	−1.1 (6.5)%	1.3 (6.0)%	−2.3 (1.0)%
15 μl/h	−2.60%	n.d.	−3.4 (5.1)%	n.d.
30 μl/h	−2.40%	0.9 (2.2) %	n.d.	n.d.
30 μl/h for 30 min + 9 ul/h for 3 days	n.d.	n.d.	−5.3 (2.5)%	n.d.
15 μl/h for 12 h + 9 ul/h for 12 h	n.d.	n.d.	−1.3(2.7)%	n.d.

Mean (s.e.m). *The manufacturer did not provide variance measurements on their flow rate accuracy. n.d.- not determined.

## References

[b1] da Silva A. A. . Chronic central nervous system MC3/4R blockade attenuates hypertension induced by nitric oxide synthase inhibition but not by angiotensin II infusion. Hypertension 65, 171–177 (2015). 25287400 10.1161/HYPERTENSIONAHA.114.03999 PMC4267912

[b2] Martineau L. & Ducharme M. B. A Chronic Arterial Cannula for Blood Sampling in Conscious, Unrestrained Rats. Contemp Top Lab Anim Sci 37, 67–72 (1998). 12456136

[b3] Ruttimann E. B., Arnold M., Hillebrand J. J., Geary N. & Langhans W. Intrameal hepatic portal and intraperitoneal infusions of glucagon-like peptide-1 reduce spontaneous meal size in the rat via different mechanisms. Endocrinology 150, 1174–1181 (2009). 18948395 10.1210/en.2008-1221 PMC2654737

[b4] Qu W. M., Huang Z. L., Matsumoto N., Xu X. H. & Urade Y. Drug delivery through a chronically implanted stomach catheter improves efficiency of evaluating wake-promoting components. J Neurosci Methods 175, 58–63 (2008). 18761374 10.1016/j.jneumeth.2008.08.002

[b5] Speed J. S., George E. M., Arany M., Cockrell K. & Granger J. P. Role of 20-hydroxyeicosatetraenoic acid in mediating hypertension in response to chronic renal medullary endothelin type B receptor blockade. PloS one 6, e26063 (2011). 22016812 10.1371/journal.pone.0026063 PMC3189228

[b6] Pawlowska D., Granger J. P. & Knox F. G. Effects of adenosine infusion into renal interstitium on renal hemodynamics. The American journal of physiology 252, F678–682 (1987). 3565578 10.1152/ajprenal.1987.252.4.F678

[b7] Mattson D. L., Roman R. J. & Cowley A. W. Jr. Role of nitric oxide in renal papillary blood flow and sodium excretion. Hypertension 19, 766–769 (1992). 1592478 10.1161/01.hyp.19.6.766

[b8] Chauhan A. . A rat model of central venous catheter to study establishment of long-term bacterial biofilm and related acute and chronic infections. PloS one 7, e37281 (2012). 22615964 10.1371/journal.pone.0037281 PMC3353920

[b9] Buommino E., Scognamiglio M., Donnarumma G., Fiorentino A. & D’Abrosca B. Recent advances in natural product-based anti-biofilm approaches to control infections. Mini Rev Med Chem 14, 1169–1182 (2014). 25553429 10.2174/1389557515666150101095853

[b10] Struyker-Boudier H. A. & Smits J. F. The osmotic minipump: a new tool in the study of steady-state kinetics of drug distribution and metabolism. J Pharm Pharmacol 30, 576–578 (1978). 29098 10.1111/j.2042-7158.1978.tb13327.x

[b11] Capozza R., Eckenhoff B. & Yum S. I. Design and performance of the implantable osmotic minipump. J Med Eng Technol 1, 281–283 (1977). 597553 10.3109/03091907709162196

[b12] Shaffery J. P., Lopez J. & Roffwarg H. P. Brain-derived neurotrophic factor (BDNF) reverses the effects of rapid eye movement sleep deprivation (REMSD) on developmentally regulated, long-term potentiation (LTP) in visual cortex slices. Neurosci Lett 513, 84–88 (2012). 22361363 10.1016/j.neulet.2012.02.012 PMC3307368

[b13] Tan T., Watts S. W. & Davis R. P. Drug Delivery: Enabling Technology for Drug Discovery and Development. iPRECIO Micro Infusion Pump: Programmable, Refillable, and Implantable. Front Pharmacol 2, 44 (2011). 21863140 10.3389/fphar.2011.00044 PMC3149148

[b14] Hyndman K. A., Musall J. B., Xue J. & Pollock J. S. Dynamin activates NO production in rat renal inner medullary collecting ducts via protein-protein interaction with NOS1. American journal of physiology. Renal physiology 301, F118–124 (2011). 21490139 10.1152/ajprenal.00534.2010 PMC3129883

[b15] Hyndman K. A. . Renal Collecting Duct NOS1 Maintains Fluid-Electrolyte Homeostasis and Blood Pressure. Hypertension 62, 91–98 (2013). 23608660 10.1161/HYPERTENSIONAHA.113.01291 PMC3901402

[b16] Xie L. . Quantitative analysis of aquaporin-2 phosphorylation. American journal of physiology. Renal physiology 298, F1018–1023 (2010). 20089674 10.1152/ajprenal.00580.2009 PMC2853310

[b17] Saito A. . A synthetic inhibitor of histone deacetylase, MS-27-275, with marked *in vivo* antitumor activity against human tumors. Proceedings of the National Academy of Sciences of the United States of America 96, 4592–4597 (1999). 10200307 10.1073/pnas.96.8.4592 PMC16377

[b18] Suzuki T. . Synthesis and histone deacetylase inhibitory activity of new benzamide derivatives. Journal of medicinal chemistry 42, 3001–3003 (1999). 10425110 10.1021/jm980565u

[b19] Bolden J. E., Peart M. J. & Johnstone R. W. Anticancer activities of histone deacetylase inhibitors. Nat Rev Drug Discov 5, 769–784 (2006). 16955068 10.1038/nrd2133

[b20] Babey M., Kopp P. & Robertson G. L. Familial forms of diabetes insipidus: clinical and molecular characteristics. Nat Rev Endocrinol 7, 701–714 (2011). 21727914 10.1038/nrendo.2011.100

[b21] Zhang L. . Trend of histone deacetylase inhibitors in cancer therapy: isoform selectivity or multitargeted strategy. Med Res Rev 35, 63–84 (2015). 24782318 10.1002/med.21320

[b22] Pili R. . Phase I study of the histone deacetylase inhibitor entinostat in combination with 13-cis retinoic acid in patients with solid tumours. Br J Cancer 106, 77–84 (2012). 22134508 10.1038/bjc.2011.527 PMC3251867

[b23] Ree A. H. . Vorinostat, a histone deacetylase inhibitor, combined with pelvic palliative radiotherapy for gastrointestinal carcinoma: the Pelvic Radiation and Vorinostat (PRAVO) phase 1 study. Lancet Oncol 11, 459–464 (2010). 20378407 10.1016/S1470-2045(10)70058-9

[b24] Kuroki M. T., Fink G. D. & Osborn J. W. Comparison of arterial pressure and plasma ANG II responses to three methods of subcutaneous ANG II administration. American journal of physiology. Heart and circulatory physiology 307, H670–679 (2014). 24993045 10.1152/ajpheart.00922.2013 PMC4187401

