# Supplementary material for: In vivo organ specific drug delivery with implantable peristaltic pumps
Source: Sci Rep. 2016 May 17;6:26251. doi: 10.1038/srep26251 (PMC4869096; doi:10.1038/srep26251)
Supplement: Supplementary Information [file srep26251-s1.pdf]

SUPPLEMENTARY NOTE 1

In vivo organ specific drug delivery with implantable peristaltic pumps

Joshua S. Speed and Kelly A. Hyndman
Cardio-Renal Physiology and Medicine, Division of Nephrology, Department of Medicine, The University of Alabama at Birmingham, Birmingham, AL 35294

ANIMAL SURGERY

All animal use and welfare adhered to the NIH Guide for the Care and Use of Laboratory Animals following a protocol reviewed and approved by the Institutional Laboratory Animal Care and Use Committee of The University of Alabama at Birmingham. Sprague Dawley, 8 week old male rats (225 g) were purchased from Harlan (Indianapolis, IN) and maintained on a 12 h light 12 h dark schedule. Rats were fed a normal salt diet (0.49% NaCl Teklad TD.96208) and water ad libitum.

For all animal surgeries, proper aseptic technique was used, and all drapes, supplies, surgery tools and gloves were sterilized. Rats were anesthetized with 2% isoflurane and given a s.c. injection of carprofen (5 mg/kg) and buprenorphine (0.1 mg/kg) to minimize pain post surgery. Just lateral to the left rib cage, hair is shaved, and the skin prepared by three alternative wipes of 10% betadine (Purdue Pharma, Stamford, CT) and 70% ethanol (in water). A small incision is made through the skin and muscle, and the kidney exposed. The adrenal gland of the left kidney was carefully freed from the upper pole of the renal capsule before the renal pedicle is ligated with 5-0 silk suture (Ethicon, Summerville, NJ) and the kidney removed. The muscle is sutured closed with 4-0 prolene suture (Ethicon) and the skin is then closed with surgical staples. The area is cleaned with 3% hydrogen peroxide in water, and the incision is given 0.25% Marcaine + 0.5% lidocaine (mixed 50/50). The animal is placed in a clean cage and allowed to recover for 7 days before the iPRECIO® surgery. Animals were closely monitored after surgery and if they lost more than 20% body weight, appeared lethargic or lost righting ability, they would've been euthanized and excluded from the study; however, in this study that was not observed, thus all animals in this study were included.

PUMP PREPARATION

Prior to the pump surgery, the pumps must be filled and programmed.

1) Keep new iPRECIO® (model smp-200, Tokyo, Japan) pumps in their sterile packaging and fill with sterile saline with a 27 gauge needle and syringe. Pierce the needle through the packaging into the pump, and fill the pump with ~1 ml of saline, such that saline will also fill the catheter. Once the pumps are activated, the gears will move into the on position and you cannot fill the catheter. It can only be filled by pumping, thus it is imperative you fill the reservoir and catheter before programming.
2) Program the pump using the iPRECIO® communication device and software. Each pump will need to be placed in the communication device 3 times: 1) to detect the pump, 2) to program the pump and 3) to activate the pump.
3) In this study, we programmed the pumps to deliver at 30 ul/h for 30 min (to maintain catheter patency during surgery), followed by 9 ul/h for 168 h (7 days). The program will tell you the approximate battery life of the pump, and keep in mind you may want to extend your study, so allow for extra time (in this study we programmed for 1 extra day).
4) Once the pumps were activated, they were placed in sterile saline in 50 ml tubes, and placed in a 37°C oven until surgery.
5) At time of the surgery, the pumps were removed from the saline, and the catheter cut to 6 cm long.
6) A small 5 mm circle of Alliedsil™ (Allied Biomedical, Paso Robles, CA) was punched with a one hole puncher, and then with a 23 gauge needle, a hole placed in the middle of the circle. Using the needle, the iPRECIO® catheter is then thread through the Alliedsil™ circle (**Fig. 1a, b**).
7) A small 1-2 cm piece of micro medical grade catheter vinyl tubing V/1 (Scientific Commodities, Lake Havasu City, AZ) was then inserted into the catheter and carefully glued (Loctite

superglue, Düsseldorf, Germany) into place (**Fig. 1b**). Caution must be taken not to use too much glue, not to get glue on the V1 catheter that will be inserted into the kidney, or glue the end of the catheter closed. Excess glue can be removed with cotton gauze. Trim the V/1 tip to 8 mm (or depending on how deep you want the catheter to enter the kidney).

8) The pump was then placed in warmed sterile saline with 1% pen/strep until implanted into the rat.

CHRONIC INTRAMEDULLARY INFUSION SURGERY

1) The rats were anesthetized with 2% isoflurane and placed with the right side of the animal facing up. A s.c. injection of carprofen (5 mg/kg) buprenorphine (0.1 mg/kg) was given to minimize pain.
2) The area just below the ribs and on the back was shaven, and disinfected with alternating wipes of betadine and 70% ethanol.
3) An incision through the skin, but not the muscle, was made and a large pocket made between the muscle and the skin. The pocket must be large enough to accommodate the pump.
4) The pump is then inserted with the reservoir facing towards the head of the rat, and sutured (4-0 prolene) into the muscle using the small loop on the right side of the pump.
5) A small incision is made through the muscle to the peritoneum, and the kidney exposed. The kidney is held away from the skin by propping it up with a sterile cotton swab (**Fig. 1d**).
6) Using a 23 gauge needle, make a small hole in the renal capsule where the catheter will be inserted being careful to remain superficial to avoid damage to the kidney that will result in excess bleeding. Remove any blood with a sterile cotton swab.
7) Drops of Vetbond™ tissue adhesive (3M, Saint Pual, MN) is sparingly applied with a 27 gauge needle syringe to the circle of Alliedsil™, and the catheter is inserted into the hole generated above. Using forceps hold the catheter in place for 2 min while the vetbond dries (**Fig. 1d**).
8) Gently return the kidney to the body, and suture the muscle wall, and staple the skin closed.
9) 0.25% Marcaine + 0.5% lidocaine (mixed 50/50) was placed on the incision site.

10) Rats are then returned to a clean cage and housed individually.

REFILLING OR EXCHANGING FLUIDS IN THE PUMPS WHILE IMPLANTED

1) Anesthetize the rats with 2% isoflurane and placed with the ventral side facing down (back is up).
2) Shave the back of the rat, where the pump resides. This will allow you to feel the refill port with your gloved finger.
3) Disinfect the area with alternating wipes of betadine and 70% ethanol.
4) With your gloved finger, feel for the curved side of the refill port of the pump (see figure 1). Move your finger from the side to the top of the pump, this is where you will stick a sterile 1 ml syringe with a 27-gauge needle. You will feel the needle hit the bottom of the refill port.
5) Also, place another gloved finger on the reservoir (Figure 1). You can feel if it is full (it will be stiff) or emptied (there will be a depression).
6) If removing the current solution, insert an empty, sterile 1 ml syringe with 27-gauge needle through the skin into the port, and slowly withdraw the solution. While doing this, place another gloved finger on the reservoir and you can feel it empty.
7) To fill the pump, insert a filled, sterile 1 ml syringe with 27-gauge needle through the skin, and place another gloved finger on the reservoir. As you dispense your volume (max 900 µl), you will feel the reservoir expand as it fills.

STERILIZING AND REUSING THE PUMPS

Successful reuse of the pumps has been achieved.

1) Once the pumps are cleaned of any obvious tissue or blood, the reservoir is filled with sterile saline. To clean the catheter, program the pump for an extra day of activity in order to pump sterile saline through the catheter. Once the pumps have stopped pumping, keep the reservoir full with sterile saline to maintain patency.

2) Next place the pumps in a beaker of 7 g haemo-sol enzymatic cleaner (Haemo-Sol International, Baltimore, MD) per 500 ml of diH_2O for 72 h at room temperature.
3) The pumps are then washed in sterile deionized water and placed in 2% glutaraldehyde for 12 h at room temperature.
4) Next place the pumps in sterile saline + 100 U/ml Penicillin + 100 µg/ml Streptomycin until next use.
5) When ready to reuse, take the pumps and place them in fresh sterile saline + 100 U/ml Penicillin + 100 µg/ml Streptomycin, refill with fresh saline and go through programming steps again. Once the pump is detected, programmed and activated, return the pump to the saline + 100 U/ml Penicillin + 100 µg/ml Streptomycin and place in 37°C oven until surgery. With this method, there was no obvious infection in the animals.

DELIVERY OF dDAVP OR MS275

In this study, there were 3 experimental groups: 1) saline or vehicle controls, 2) vasopressin receptor 2 agonist, dDAVP, 3) Class 1 histone deacetylase inhibitor, MS275.

Protocol 1: dDAVP

1) Two days after pump surgery, the rats were randomized to either saline control or dDAVP treatment groups, anesthetized with 2% isoflurane, and the hair shaven off the back of the animal, where the pump resides.
2) The skin was disinfected with alternating wipes of betadine and 70% ethanol. The refill port was felt through the skin, and with a 27 gauge needle syringe the remaining ~400 ul of saline withdrawn from the pump.
3) The pump was then refilled percutaneous with a 27 gauge needle syringe filled with either sterile saline (control) or 1.1 ng/µl dDAVP (Sigma-Aldrich, St. Louis, MO) in sterile saline.

4) The skin was then cleaned with 70% ethanol and the rats placed individually into metabolic cages. Rats were placed on a high salt diet (4.0% NaCl, Teklad TD.92034). Food and water intake and urine production were recorded. Urine samples were spun 1000 g for 10 min, snap frozen and stored at -80°C until analysis.
5) Urine was collected every 24 h for 4 days.
6) On the 4th day the rats were anesthetized for plasma and tissue sample collection, which were stored at -80°C until analyzed. The rats were euthanized immediately following, and pumps were carefully removed, cleaned of any tissues and placed in saline.

Protocol 2: MS275

1) Similar to above, after 2 days recovery from pump surgery, a separate group of rats were infused with either vehicle (30% DMSO in saline) or 1 mg/kg/day MS275 (30% DMSO and 70% saline) (Cayman Chemical, Ann Arbor, MI).
2) Rats were placed on a high salt diet (4.0% NaCl, Teklad). Food and water intake and urine production were recorded. Urine samples were spun 1000 g for 10 min, snap frozen and stored at -80°C until analysis.
3) Urine was collected every 24 h for 4 days.
4) On the 4th day to rats were euthanized and plasma and tissue samples collected and stored at -80°C until analyzed. Pumps were carefully removed, cleaned of any tissues and placed in saline.

WESTERN BLOTS AND HISTONE EXTRACTION.

Inner medullae from Protocol 1, were lysed in 10 vols/ wt of lysis buffer + protease inhibitors and phosphatase inhibitor cocktail (Thermo, Waltham, MA) as previously described[1], and spun at 6,500 x g for 10 min to pellet nuclei. The supernatant (herein referred to as lysate) from this spin was used to determine expression of AQP2 and phosphorylated AQP2.

Outer medulla and Cortex from Protocol 2, were lysed with lysis buffer + protease inhibitors, and phosphatase inhibitor cocktail, and centrifuged at 6,500 x g for 10 min at 4°C to pellet nuclei. Histones were then extracted from the nuclear pellet by acid extraction with 5 volumes of 0.2 N hydrochloric acid, overnight at 4°C. The histone sample was then spun at 6,500 g for 10 min at 4°C to pellet debris, and the protein concentration of the histone supernatant and original lysate determined by Bradford assay (Quickstart®, Biorad, Carlsbad, CA), and samples stored at -20°C until used in Westerns as previously described[1, 2]. Antibodies used in the study were Phosphorylated AQP2 S261, S264 or S269 (PhosphoSolutions, Aurora, CO), total AQP2 (SC-9882, Santa Cruz Biotechnology, Dallas, TX), acetylated H3 (#06-599, EMD Millipore, Billerica, MA) and total H3 (06-755, EMD Millipore). Densitometry (arbitrary units) was determined using Image Studio (Li-COR Biotechnology, Lincoln, NE), by an investigator blinded to the treatments.

Urine and plasma electrolytes.

Urine and plasma osmolality were measure by a vapor pressure osmometer (Elitech Group Solutions, Princeton, NJ). Ion selective electrodes were used to determine Na^+, K^+ and Cl^- concentrations (Easylyte, Medica, Bedford, MA).

1. Hyndman, K.A., Musall, J.B., Xue, J. & Pollock, J.S. *American journal of physiology. Renal physiology* **301**, F118-124 (2011).
2. Hyndman, K.A. et al. *Hypertension* **62**, 91-98 (2013).

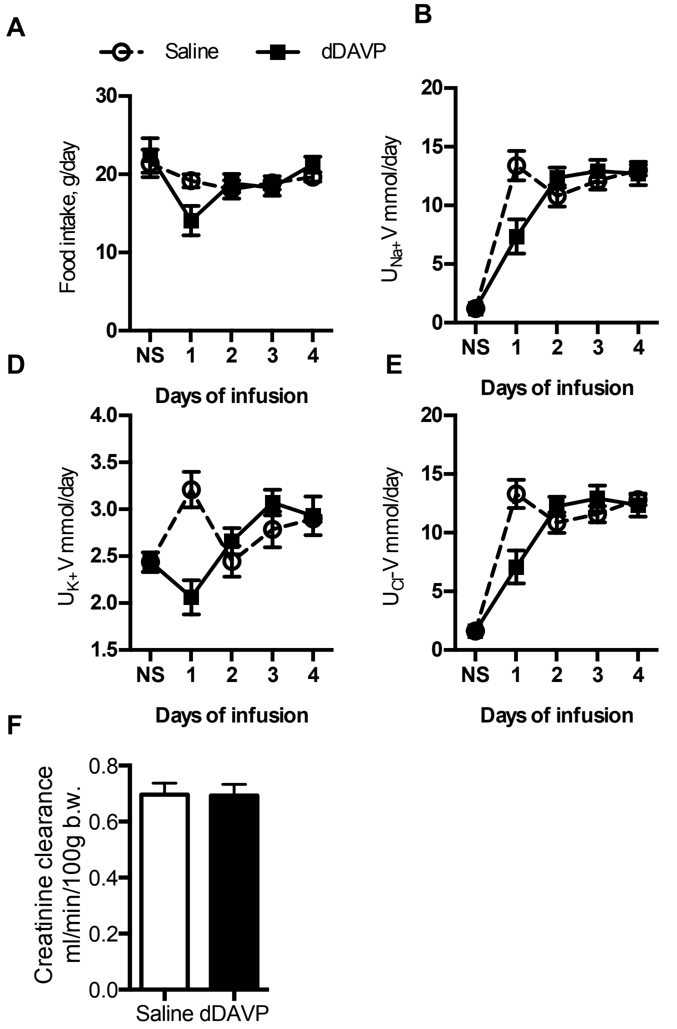

Supplementary Figure 1| Chronic intramedullary interstitial infusion of saline or dDAVP with implantable peristaltic pumps. Mean ± s.e.m. Rats were switched from a normal salt (NS) to high salt diet plus intramedullary infusion. dDAVP intramedullary (solid line), interstitial infusion did not significantly affect (**a**) food intake (Repeated Measures, Two-Factor ANOVA, $P_{group} > 0.05$, $P_{diet} = 0.008$, $P_{interaction} = 0.08$, $P_{subjects\ matching} = 0.4$), (**b**) sodium excretion ($P_{group} > 0.05$, $P_{diet} < 0.05$, $P_{interaction} < 0.05$, $P_{subjects\ matching} = 0.05$), (**c**) potassium excretion ($P_{group} > 0.05$, $P_{diet} < 0.05$, $P_{interaction} < 0.001$, $P_{subjects\ matching} = 0.3$), (**d**) chloride excretion ($P_{group} > 0.05$, $P_{diet} < 0.001$, $P_{interaction} < 0.001$, $P_{subjects\ matching} = 0.06$) compared to saline infused control rats (Dotted line). (**f**) creatinine clearance determined on day 4 of the experiment, was similar between the groups (unpaired, two tailed, Student's t-test, p =0.95) N= 4/group.

A **B**

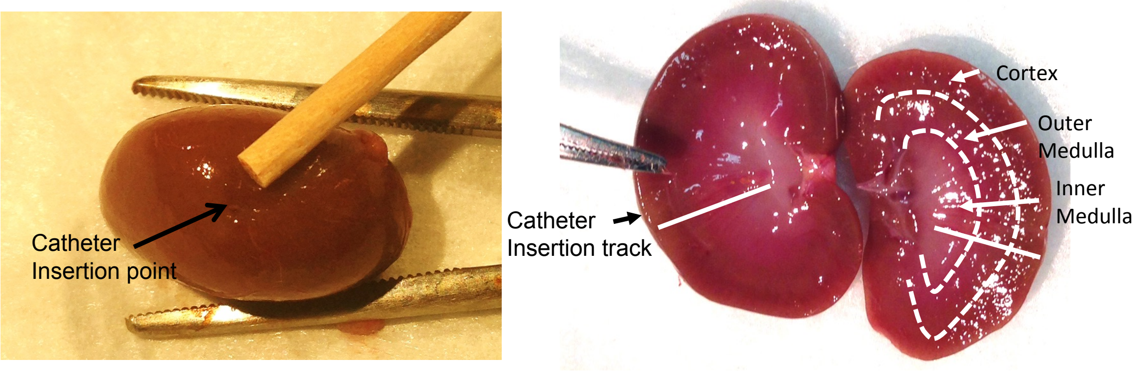

Supplementary Figure 2| Kidneys dissected post infusion. (**a**) the wooden stick points to the catheter insertion point in the kidney. (**b**) Gross dissection of the kidney reveals a proper track of insertion from the cortex to the junction of the outer and inner medulla. The white line is just distal to the track. The dashed line outlines the cortex, outer and inner medulla sections of the rat kidney.
